# Practical Chemistry and Pharmacy

**Published:** 1894-06

**Authors:** 

**Affiliations:** LaGrange, Ga.


### *SELECTIONS AND ABSTRACTS.*

### PRACTICAL CHEMISTRY AND PHARMACY.

EDITED BY HENRY R. SLACK, M.D., PH.M., LAGRANGE, GA.

### INCOMPATIBILITY OF CALOMEL AND POTASSIUM BROMIDE.

L. W. Thompson recently had occasion to compound a prescription calling for potassium bromide and calomel. As soon as the two ingredients were triturated together in a mortar, the mixture began to darken, and on the addition of water the powder turned grayish black. It was subsequently found that if the potassium bromide was powdered and then dried so as to remove interstitial moisture before being mixed with the calomel, there was no darkening, but the change took place instantly on the addition of moisture. The author tried several experiments, and found that the darkening is due to the separation of metallic mercury from the calomel, as explained by the following equation:

$$2\,Hg\,Cl + 4KBr = (Hg\,Br_2\,2\,K\,Br) + Hg + 2\,K\,Cl.$$

The prescription, therefore, is an example of a dangerous incompatibility, as the mercuric salt found is a powerful poison. There is no method by which the decomposition can be avoided, and calomel and potassium bromide should therefore never be prescribed together.—*Chem. and Druggist.*

The same author also points out the incompatibility of calomel and tincture of iodine, especially the tincture of iodine of the B. Ph., which contains potassium iodide and forms biniodide mercury, the reaction leaving

$$2\,Hg\,Cl + KI = Hg\,I_2\,2\,KI + Hg + 2K\,Cl.$$

### TO ESTIMATE ALKALOIDA BY MEANS OF POTASSIO-MERCURIC IODIDE.

Grandrall and Lajoux (*Pharm. Centhral*) proceed in the following manner: One hundred grammes of the plant are finely pulverized, triturated with the same quantity of lead acetate, and percolated with six hundred grammes of water. The lead acetate precipitates the organic acids, while the alkaloids dissolve as acetates. There are also found insoluble compounds of coloring and extractive matter with lead, and most of the albuminoids are precipitated. The filtrate is mixed with an excess of sulphuric acid; the lead sulphate is filtered off, and the alkaloids are precipitated by an excess of solution of potassio-mercuric iodide. The precipitate is washed and may be decomposed by either of the following methods: (1) By adding potassium cyanide and some soda-lye, and shaking out with ether (the addition of a small quantity of oil causes speedy separation); or (2) by adding a slight excess of sodium-sulphide solution, acidulating after awhile with sulphuric acid filtering, mixing the filtrate with potassa-solution, and shaking the alkaloid out with ether containing oil or with some other appropriate fluid.

---

### Artificial Citric Acid.

We announced some months ago the claim that citric acid had been obtained by the fermentation for glucose. This transformation was brought about by two species of fungi which had escaped previous observation, their discoverer being a Hanoverian botanist.

A further report which has come to our attention shows that the process which has been patented in several countries is being experimented with on a large scale, and the prospects are highly favorable that the artificial product can be made much more cheaply than the natural.

If this prove true, the lemon growers will, of course, suffer seriously, as fruit unmerchantable as such is utilized in the preparation of the acid, the peel furnishing at the same time the well known lemon oil.

Perhaps, however, the growers, deprived of one source of profit, may manage to make up the loss by adding to the price of the oil. No process has yet been discovered for preparing it artificially, and the cost of production will necessarily have to be met.—*Druggists' Cir. and Chem. Gazette.*

### MAGNESIUM SULPHATE SUBCUTANEOUSLY.

Dr. J. H. Wade studied the purgative action of sabcutaneous injections of magnesium sulphate, on forty-six patients, injecting quantities of a two per cent. solution representing 1.86–4.5 grains (12–30 ctg.) of the salt. Two small doses at small intervals proved more effective than a single large dose. The injections were made on the left arm, and produced no ill after-effects; sixty-seven out of the one hundred were successful. An evacuation followed five times, and two stools ten times. The shortest interval between the injection and its effect was three hours, the longest fourteen hours. This method of administering magnesium sulphate would seem to be indicated in gastritis, abdominal surgery, and in conditions of unconsciousness.—*Med. News.*

---

### PERMANENT MOUNTS OF URINARY DEPOSITS.

Von Frisch states (*Zeit. Oest. Apoth. Ver.*) that by the following method he is able to mount and preserve, microscopically, specimens of urinary deposits which have kept well for three years. The medium he employs is a glycerin jelly of one part of gelatin, four parts of glycerin, and two parts of distilled water. A drop of the deposit is placed on the glass cover and partially dried, a drop of the gelatin solution is placed on a warm slide, and the cover glass with the deposit carefully placed over it. The method is equally available for crystalline or organized sediments; for the latter, however, the author recommends that the gelatin should be colored with fuchsin, when the organized elements are gradually stained by the dye.

---

### SALICYLATES IN RHEUMATISM.

Dr. Han says that if the salicylates do not yield good results in cases of rheumatism in from four to six days, their use should be discontinued, and other drugs tried, as it is not likely that any good results will follow their further use. He does not think that they should be administered in large doses three or four times a day. He has had the best results follow from the frequent administration of small doses.—*Druggists' Circular.*

REMOVING aniline stains from the skin by acids or hypochlorites, is frequently impracticable, owing to its consequence. Unna, therefore, proposes to wash the hands, first with a five per cent. sodium-chloride solution, then with a five per cent. solution of hydrogen peroxide, and to rub them with linen dipped in alcohol.—*Pharm. Ztg.*

